# Recent Developments in Enteric Bacterial Vaccines

**DOI:** 10.3390/microorganisms9081604

**Published:** 2021-07-28

**Authors:** Sjoerd Rijpkema, Barbara Bolgiano

**Affiliations:** The National Institute for Biological Standards and Control (NIBSC), South Mimms EN6 3QG, UK; Barbara.Bolgiano@nibsc.org

In this issue, we present promising developments in the field of bacterial enteric vaccines. For the last three decades, the development of new enteric vaccines has played Cinderella to the three ugly sisters Malaria, HIV and TB. Public health programs for enteric vaccines historically suffered from underfunding, despite enteric pathogens being major killers of infants in areas with a high burden of disease, despite the World Health Organization (WHO), non-governmental organizations and aid agencies committing resources to preventing cholera, typhoid, invasive non-typhoidal salmonellosis and shigellosis in low-income and middle-income countries (LMICs). A change came in 2010, when vaccination was chosen as the main intervention strategy to combat a major cholera epidemic in Haiti. This put vaccines in the spotlight as a control measure for enteric diseases. In 2013, the WHO established the stockpile for inactivated oral cholera vaccines (OCVs) to meet the global demand for this vaccine [1]. To date, more than 13 million doses have been distributed from the OCV stockpile, which is replenished with the help of vaccine manufacturers and WHO collaborating centers, who independently verify the quality of these vaccines. This effort is a major part of the WHO campaign to eliminate cholera outbreaks worldwide by 2030.

The world received a further boost by the campaigns with newly developed rotavirus vaccines for infants, which have had a tremendous impact on the morbidity and mortality of this diarrheal disease, lowering the cost to public health as well as limiting the economic impact of the disease. The involvement of the Bill and Melinda Gates Foundation (BMGF) provided crucial financial support and guidance for these programs and also expedited the development and licensing of new bacterial vaccines for enteric diseases, exemplified by the incorporation of new typhoid conjugate vaccines (TCVs) in the public health programs of LMICs.

The WHO collaborating centers played a role in smoothing regulatory hurdles associated with the quality control of the OCVs and new TCVs. In 2009, the WHO Expert Committee on Biological Standardization approved a major standardization program for new enteric vaccines with the assistance of one vaccine developer, Novartis Vaccines for Global Health (now Glaxo Vaccines for Global Health; GVGH). This program also benefited from the input of the WHO, the Coalition against Typhoid, PATH, BMGF, the International Vaccine Institute (IVI), the Oxford Vaccine Group (OVG) and others. This network delivered WHO workshops to train local laboratory staff, WHO international standards for batch release assays and ‘open access’ in vitro diagnostic tests for comparing the performance of vaccines used in different clinical trials or field settings. In addition, written WHO guidelines were produced such as the recently updated guideline on the safety and quality of TCVs [2].

Currently, WHO international standards are being developed for Shigella vaccines, inactivated oral cholera vaccines and vaccines for invasive non-typhoidal salmonellosis. It is hoped that these reference reagents will aid the development of batch release assays and thereby the manufacture and deployment of these vaccines.

The impact of the introduction of new TCVs and OCVs will slow the dissemination of antimicrobial resistance and will impact on the spread and evolution of antibiotic resistance organisms amongst humans and animal species used for food production. It will help to reduce outbreaks in areas subject to flooding and with an insecure clean water supply and protect the health of communities from life-threatening and debilitating diarrheal disease and enteric fever.

In this issue, the contributions from leading authors provide the following:

- A comprehensive update on the status of more than thirty candidate vaccines to protect against diarrheal disease (Walker et al.);
- Insight into serum markers of immunogenicity (Jones et al.);
- Studies highlighting the implementation of assays to improve the safety of vaccines containing outer membrane vesicles (Carson et al.);
- Studies describing methods which can be used to show the thermal stability of vaccines outside the cold chain (Gao et al.);
- In silico engineering of the fimbrial tip adhesin holds promise for new improved subunit vaccines to combat enterotoxigenic *Escherichia coli* infections (Liu et al.).

This collection of reports taken together will help to improve our understanding of these new enteric vaccines and ultimately expedite their acceptance and use as public health tools in resource-stretched settings.
